# RPFeaNet: Rethinking Deep Progressive Prompt-Guided Feature Interaction Fusion Network for Medical Ultrasound Image Segmentation

**DOI:** 10.3390/s26082394

**Published:** 2026-04-14

**Authors:** Lei Zhu, Yuqing Du

**Affiliations:** 1 School of Mechanics and Engineering Science, Peking University, Beijing 100871, China; 2201120077@stu.pku.edu.cn; 2 Academy of Artificial Intelligence, Beijing Institute of Petrochemical Technology, Beijing 102617, China

**Keywords:** progressive prompt, Mamba, frequency selection, ultrasound image segmentation

## Abstract

Although ultrasound image segmentation has advanced significantly with deep learning, existing methods still suffer from a lack of prior knowledge guidance, partly due to the low-contrast, speckle-noise-corrupted nature from clinical ultrasound sensors. This paper proposes a novel ultrasound segmentation framework (RPFeaNet) that extracts progressive prompts from a low-to-high level prompt generation mechanism. Furthermore, the high-level prompt-guided feature interaction module (HPGFIM) fuses progressive prompt via Mamba blocks and stage-wise condition injection. The dynamic selective-frequency decoder (DSFD) combines dynamically selecting a strategy with the fusion of high-frequency details to suppress noise and refine edge details. Extensive experiments on six datasets demonstrate that RPFeaNet achieves state-of-the-art performance compared to existing methods, validating its strong generalization and robustness across diverse clinical ultrasound scenarios.

## 1. Introduction

Medical imaging sensors, particularly medical ultrasound sensors, have been increasingly advanced by the rapid development of image segmentation [1] and deep learning [2], effectively improving the diagnostic value of data acquired by medical ultrasound sensors. Ultrasound image segmentation has emerged as a pivotal technology in medical image processing, leveraging automatically segmented boundary information for the diagnosis of various organs and tissues such as carotid plaques [3], carotid arteries [4], breast lesions [5], and nerves [6], among others. Through image segmentation, sensors can more effectively identify regions of interest, and this capability can be significantly enhanced to achieve superior accuracy and reliability in clinical diagnosis. However, ultrasound images are inherently degraded by speckle noise [7], low contrast, and artifacts such as acoustic shadows, which lead to ambiguous lesion boundaries and greatly increase segmentation difficulty [8]. Regions with drastic grayscale changes, such as edges and shadowed areas, are particularly prone to segmentation errors. Unlike X-ray or magnetic resonance imaging, ultrasound imaging is based on the reflection and scattering of ultrasonic waves. This distinct imaging mechanism exacerbates the aforementioned issues, making it harder to distinguish tissue boundaries. Additionally, the lack of large-scale, high-quality annotated data [9] further hinders the development of accurate segmentation models. Thus, these factors make ultrasound image segmentation a highly challenging task in medical image analysis.

Many existing deep learning-based methods have been adopted for various ultrasound image segmentation tasks. Various network architectures, including convolutional neural networks (CNNs) [10], vision Transformers (ViTs) [11], visual state space models (VSS) [12], generative adversarial networks (GANs) [13], and other advanced designs have been applied to ultrasound image segmentation. However, these representative architectures only focus on general principles of feature extraction rather than the inherent characteristics of ultrasound medical images themselves. Fully connected networks, such as UNet, and their various variants [14] have achieved remarkable success in ultrasound image segmentation through feature-level or attention-level improvements. Swin Transformers [15] have been applied to breast tumor ultrasound images due to their ability to model long-range dependencies. Deepak et al. [16] proposed using an attention mechanism to handle broken boundaries, which are common in ultrasound images. Lyu et al. [17] exploited spatial and channel attention to help the model focus on edge information. While these methods follow the methodology of advanced feature learning, they mainly rely on generic deep learning paradigms (e.g., multi-scale feature fusion [18], self-attention aggregation [19]) rather than integrating domain-specific knowledge of ultrasound images. Specifically, such methods are usually derived from statistical characteristics of training data or heuristic structural designs, lacking the ability to adapt to the inherent properties of ultrasound images.

In order to discover the inherent guidance of medical images, increasingly more works have adopted the prior-guided prompt network for medical image segmentation tasks. Prompt–domain representation learning has been incorporated into not only convolutional neural networks (CNNs) but also Transformers, serving as a core module to enhance feature discriminability in ultrasound image segmentation. However, simple text-driven prompt methods using contrast language–image pretraining (CLIP) have actually exacerbated the domain gap, especially in ultrasound image. Single text guidance has been applied into ultrasound image segmentation; Chen et al. [20] used text prompt-guided multiple organ segmentation, and Spiegler et al. [21] explored the text-driven adaptation of segment anything model (SAM) without manual geometric prompts. Gowda et al. [22] designed different adapters used to refine the representation of low-contrast ultrasound images. To enhance boundary perception, Zhang et al. [23] used the DINOv3 pretrained foundation model with multi-scale frequency extraction and alignment to enrich spatial coherence. Despite the progress of prompt–domain knowledge (edge, boundary, spatial information, etc.) in the current model, these methods rely on explicit modeling manners by fusing conventional network architectures. However, pervasive speckle noise, low contrast, and shadow artifacts often cause target boundaries to become blurred, broken, or even indistinguishable from the background in ultrasound imaging. Thus, their predictions tend to be unstable for low-saliency objects, especially small targets and weak boundaries. This raises a critical question to discuss: Manually designed prompts struggle to effectively guide and optimize feature extraction. This inherent limitation has driven the emergence of numerous automatic prompt generation methods for medical image segmentation [21], aiming to enhance the model’s ability to capture high-level semantic features of objects. Without explicit prompts, they [24,25] generated automatic prompts for ultrasound image segmentation. Various prompts [26] have been proposed for object segmentation refinement. However, they lack a structured way to integrate multi-level prior information, leading to unstable performance on diverse ultrasound datasets.

**Figure 1:**
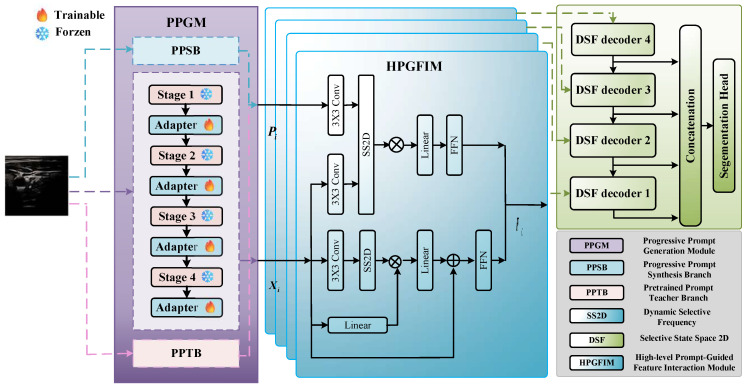
Overall architecture of the proposed RPFeaNet for medical ultrasound image segmentation. It consists of three core components: (1) The progressive prompt generation module (PPGM) with frozen backbone stages and trainable adapters, which generates multi-level spatial prior prompts $P_{i}$ from the input image. (2) The high-level prompt-guided feature interaction module (HPGFIM), which fuses the backbone feature $X_{i}$ with the prompts $P_{i}$ via Selective State Space 2D (SS2D) blocks to enhance target-aware feature representation. (3) The dynamic selective-frequency (DSE) decoder stack, which refines the fused features in the frequency domain and produces the final segmentation map through a concatenation head.

To address the above critical issue, we aim to design a method that can enrich multi-level spatial prior information for low-contrast ultrasound images by a progressive prompt-driven perspective shown in Figure 1. We design a progressive prompt generation module to capture low-to-high level information for ultrasound images. This type of progressive prompt is expected to guide the model to gradually understand the characteristics of low-contrast ultrasound images, rather than relying on simple text descriptions. Compared with the existing works, it is not a simple combination of multiple discrete prompts, but a logically hierarchical elevation that mines multi-level spatial priors from the SAM2 Hiera [27], patch-level encoder and CLIP in a low-to-high progressive manner for ultrasound images. Low-level structural priors from the frozen CLIP encoder, middle-level dense priors from the patch-level encoder, and high-level semantic priors from SAM2’s hierarchical feature encoding. This forms a coherent prior system for ultrasound images instead of a disjointed prompt ensemble. Furthermore, the high-level prompt-guided feature interaction module (HPGFIM) is designed for guiding the model to focus on the essential shape and texture feature. To this end, we design this module to take progressive prompts as explicit conditioning signals and implement high-level stage-aware guidance via conditioning injection. This integration tailors Mamba/SS2D for capturing the shape and texture features of ultrasound targets, rather than merely performing a naive feature concatenation of prompts and backbone features. Finally, to bridge the gap between an encoder and decoder, the dynamic selective-frequency decoder is proposed based on selective-frequency decomposition and a multi-level frequency gating mechanism to enhance the ability of the spatial-frequency conversion pattern between high-level semantic features and low-level details. Overall, the main contributions can be summarized as follows:

- The progressive prompt generation module is used to effectively enrich multi-level spatial prior information for low-contrast ultrasound images from a progressive prompt-driven perspective.
- The high-level prompt-guided feature interaction fusion module is designed to achieve progressive prompt interaction fusion via visual mamba network and stage-wise conditioning injection.
- The extensive experiments on six ultrasound image benchmark datasets demonstrate that our proposed method, RPFeaNet, achieves superior performance over comparative state-of-the-art methods.

The remaining sections of this paper are organized as follows. Section 2 reviews relevant research in vision Transformer-based ultrasound image segmentation and prompt-based ultrasound image segmentation. Section 3 describes the proposed RPFeaNet framework in detail. Section 4 presents the experimental setup, evaluation metrics, and comparative results against state-of-the-art approaches, followed by ablation studies and qualitative analyses. Finally, Section 5 concludes this paper and discusses future work.

## 2. Related Works

### 2.1. CNN/Transformer for Ultrasound Image Segmentation

Early attempts to introduce Transformers into ultrasound image segmentation primarily focused on integrating Transformer modules into convolutional neural network (CNN) architectures to compensate for the limited receptive field of convolution operations. Chen et al. [28] were among the first to demonstrate that incorporating Transformer components into CNN backbones can effectively enhance global context modeling, leading to improved structural consistency compared with purely convolutional approaches. Following this line of research, more structured hybrid CNN–Transformer architectures were proposed to better exploit the complementary strengths of both paradigms. He et al. [29] introduced Transformer encoder blocks (TEBlocks) into the encoder to explicitly model long-range dependencies, while interleaving convolutional layers and Transformer modules in a hybrid stem. Subsequent works further refined hybrid designs by enhancing feature representation and fusion strategies. For example, HAU-Net [30] embedded Transformer blocks into U-shaped skip connections to strengthen cross-layer semantic interaction. While the aforementioned hybrid CNN–Transformer methods alleviate some limitations of purely convolutional networks, they still rely heavily on convolutional structures for feature extraction. To further explore the potential of Transformer-based models in vision tasks, researchers began to investigate vision Transformers as standalone backbones. However, directly adapting Transformers from natural language processing to vision tasks poses significant challenges, mainly due to the high spatial resolution of images and large-scale variations of visual entities.

Motivated by the success of the Swin Transformer in general vision tasks, subsequent studies extended hierarchical Transformers to medical image segmentation. In particular, Swin-Unet [31] introduced a pure Transformer-based U-shaped encoder–decoder architecture that replaces convolutional operations with hierarchical Swin Transformer blocks. In ultrasound image segmentation, hierarchical Transformers were further combined with CNNs to enhance both local detail preservation and global semantic modeling. Yang et al. [32] extended this idea by introducing a CNN–Swin pyramid architecture to better capture multi-scale lesion characteristics. More recent studies have explored parallel dual-branch architectures, where CNN and Swin Transformer encoders operate concurrently. These developments mark a transition from sequential hybrid designs to parallel and hierarchical CNN–Transformer frameworks in ultrasound segmentation.

### 2.2. Prompt-Guided Ultrasound Image Segmentation

While text-based prompting technology is common in medical image segmentation tasks, the lack of image/label and anatomical priors degrades the performance of prompt-based methods when applied to ultrasound image segmentation. Ref. [21] explored the text-driven adaptation of the segment anything model (SAM) without the need for manual geometric prompts. Gowda et al. [22] designed different adapters to refine the representation of low-contrast ultrasound images. Without explicit prompts, Yin et al. [24] generated automatic prompts for breast lesion segmentation. Tian et al. [33] proposed a self-prompting mechanism to capture temporal features for carotid artery diagnosis. Zhao et al. [34] avoided reliance on manual prior guidance and proposed an uncertainty-driven edge prompt to enrich medical image features. However, all the aforementioned existing works relied only on a single prompt mechanism to enhance the model’s learning ability. A single prior is insufficient to overcome the limitations of low-contrast ultrasound images. Thus, we further address this gap by adopting a progressive prompt mechanism to further boost the learning capability for ultrasound image segmentation. The Table 1 shows the comparison of RPFeaNet with representative ultrasound image methods from different perspectives.

## 3. The Proposed RPFeaNet

### 3.1. The Overview of RPFeaNet

Given an ultrasound image $I\in R^{H\times W\times 3}$, we propose RPFeaNet, a progressive prompt-guided feature interaction fusion segmentation network designed to mitigate speckle noise, low contrast, and boundary ambiguity. The key idea is to (1) generate reliable progressive prompts from global-to-local perspectives, (2) perform high-level prompt-guided interaction and fusion on multi-scale features, and (3) decode features by dynamic selective-frequency modeling to recover sharp boundaries. As shown in Figure 1, RPFeaNet follows an encoder–decoder paradigm and consists of three major components: (i) the progressive prompt generation module (PPGM), (ii) the high-level prompt-guided feature interaction module (HPGFIM), and (iii) the dynamic selective-frequency decoder (DSFD). Specifically, the progressive prompt generation module (PPGM) is elaborated in Section 3.2, which serves as the core component for generating high-quality dense prompts. PPGM comprises three collaborative branches, namely the pretrained prompt teacher branch (PPTB), the prior prompt synthesis branch (PPSB), and the lightweight feature extractor (LFE), corresponding to low-level, middle-level, and high-level knowledge, respectively. To clarify their relationships, PPTB provides stable structural priors via a frozen Transformer backbone, acting as foundational low-level guidance. PPSB builds upon this prior by synthesizing patch-level cues and dense embeddings, forming the middle-level knowledge. Subsequently, LFE extracts high-level semantic representations from a lightweight encoder. These three branches are not independent but interact through a progressive fusion strategy, in which knowledge is gradually integrated from low-level to high-level representations. The final fused dense prompts generated by PPGM are then fed into the high-level prompt-guided feature interaction module (HPGFIM), where they guide the interaction of multi-scale features to further enhance segmentation performance.

These PPTB, PPSB, LFE branches in PPGM enable a coarse-to-fine prompting strategy that significantly enhances model generalization across low-contrast datasets. Here, we describe a data flow of the overall pipeline.

1. PPTB: Given input *I*, a frozen Transformer backbone outputs stable structural prompts $P_{\mathrm{tea}}$.
2. PPSB: Based on *I*, patch embeddings are synthesized into dense prompts $P_{\mathrm{dense}}$.
3. LFE: A lightweight encoder extracts semantic features *X*;

The three types of knowledge are integrated as $P_{\mathrm{fusion}}=\mathrm{Fuse}\left(P_{\mathrm{tea}},P_{\mathrm{dense}},X\right)$.

The final fused dense prompts $P_{\mathrm{fusion}}$ generated by PPGM are then fed into the high-level prompt-guided feature interaction module (HPGFIM), where they guide the interaction between multi-scale features:

$$F_{\mathrm{enhanced}}=\mathrm{HPGFIM}\left(X,P_{\mathrm{fusion}}\right),$$

to enhance segmentation performance. The details can be found in the following sections.

### 3.2. Progressive Prompt Generation Module (PPGM)

Unlike natural or general medical imaging, feature extraction from ultrasound images is fundamentally constrained by intrinsic low contrast and speckle noise. Consequently, effective representation learning for ultrasound image must go beyond standard visual encoding. From our insights, relying solely on pixel representation does not facilitate subsequent tasks; it must actively compensate for the structural degradation caused by low contrast. To address this challenge, we introduce a progressive prompting strategy, which refines structural priors to overcome the inherent limitations of medical ultrasound images.

We propose the progressive prompt generation module (PPGM), designed to generate high-fidelity dense prompts for medical image segmentation without requiring manual intervention.

1. Lightweight Feature Extractor (LFE): A lightweight encoder produces the knowledge from a pretrained network termed “High-Level Knowledge”.
2. Prior Prompt Synthesis Branch (PPSB): An encoder–generator pipeline that synthesizes patch-level cues and dense embedding termed “Middle-Level Knowledge”.
3. Pretrained Prompt Teacher Branch (PPTB): A frozen Transformer backbone that utilizes trainable prompt tokens to provide stable representation evolution and strong structural priors, termed “Low-Level Knowledge”.

#### 3.2.1. Lightweight Feature Extractor (LFE)

Given an input image $I\in R^{H\times W\times 3}$, we adopt a hierarchical visual Transformer encoder called Hiera [27] that outputs a four-stage feature pyramid:

(1) $$\left\{X_{1},X_{2},X_{3},X_{4}\right\}=\Phi_{\mathrm{enc}}\left(I\right),$$

where $X_{k}\in R^{C_{k}\times \frac{H}{2^{k}}\times \frac{W}{2^{k}}}$ denotes the feature map at the *k*-th stage (with progressively reduced spatial resolution and enriched global context).

To enable parameter-efficient domain adaptation, we freeze the pretrained encoder trunk and insert lightweight adapters before each block:

(2) $${\hat{X}}_{k}=X_{k}+A_{k}\left(X_{k}\right),\;k\in \left\{1,2,3,4\right\},$$

where $A_{k}\left(\cdot \right)$ denotes a stage-wise adapter. Since the hierarchical encoder contains a substantial number of parameters, we freeze the encoder trunk and insert adapters before each multi-scale block for parameter-efficient fine-tuning. Each adapter is implemented as a two-layer MLP with GeLU nonlinearity:

(3) $$A_{k}\left(X\right)=\mathrm{GeLU}\;\left(W_{↑}^{\left(k\right)}\;\mathrm{GeLU}\;\left(W_{↓}^{\left(k\right)}X\right)\right),$$

where $W_{↓}^{\left(k\right)}$ and $W_{↑}^{\left(k\right)}$ are the down-projection and up-projection linear mappings, respectively.

#### 3.2.2. Pretrained Prompt Teacher Branch (PPTB)

To provide stable structural guidance and high-level semantic priors, we introduce the pretrained prompt teacher branch (PPTB), which utilizes a fine-tuned enhanced CLIP visual encoder [36]. While standard foundation models are pretrained on natural images, their direct application to ultrasound (US) imaging is hindered by the domain gap and low-contrast characteristics. We keep the CLIP-ViT backbone $\Phi_{CLIP}$ frozen and insert a sequence of learnable visual prompts $P={\left\{p_{i}\right\}}_{i=1}^{N}$ to bridge this gap.

The PPTB processes it through the prompt-tuned CLIP encoder to generate a stable teacher representation $P_{tea}$. The interaction within the Transformer layers is defined as follows:

(4) $$P_{tea}=\Phi_{\mathrm{CLIP}}\left(\left[x_{i}^{l-1},P^{l-1}\right]\right)$$

where $T_{l}$ denotes the *l*-th Transformer layer, and $x_{i}$ represents the image features by optimizing only the prompt tokens P while keeping the backbone weights fixed.

#### 3.2.3. Prior Prompt Synthesis Branch (PPSB)

The prior prompt synthesis branch (PPSB) refines images into actionable prompts in a coarse-to-fine manner. It first processes initial patch-level cues and progressively transforms them into dense prompt embeddings aligned with the latent image space, enabling robust prompt learning without the need for manual point or box annotations. The details are presents as follows.

(5) $$x_{i}=\mathrm{PatchEmbed}\left(I\right)\in R^{N\times D},$$

where *N* denotes the number of patches and *D* is the embedding dimension.

The patch embedding is processed by a hybrid backbone with interleaved convolutional and Transformer components. Specifically, local spatial features are extracted through convolutional layers, normalization and linear projection. Moreover, a multi-head self-attention (MSA) module is subsequently applied:

(6) $$h=\mathrm{MSA}(\mathrm{Linear}\;\left(\mathrm{LayerNorm}({\mathrm{Conv}}_{2}\;\left({\mathrm{Conv}}_{1}\left(x_{i}\right)\right))\right)),$$

where the resulting embedding h simultaneously encodes fine-grained local details and global contextual information.

Specifically, within the given image embedding h, the function δ(·) performs patch merging to produce patch-level logits ${\hat{p}}_{pat}$:

(7) $${\hat{p}}_{pat}=\delta \left(h\right)$$

δ(·) is implemented using lightweight 2×2 stride-2 convolutions with a batch normalization layer, followed by a 1×1 convolution for channel compression. This stage provides a coarse but robust prompt prior. Furthermore, to obtain dense prompts, we convert patch logits ${\hat{p}}_{pat}$ to probability function σ(·) and progressively lift them to the feature resolution:

(8) $$p_{pat}=\sigma \left({\hat{p}}_{pat}\right)$$

(9) $$p_{pat}^{↑}=\mathrm{Interpolate}\left(p_{pat}\right)$$

(10) $$P_{dense}=\psi \left(p_{pat}^{↑}\right)$$

where $P_{dense}$ denotes the feature of PPSB, and ψ(·) denotes the prompt encoder, implemented by interpolation (for memory efficiency) and 1×1 convolutions to align the prompt embedding dimension to that of h.

### 3.3. High-Level Prompt-Guided Feature Interaction Module (HPGFIM)

In this way, PPGM progressively synthesizes dense prompt embedding $P_{dense}$, which can be directly fused with $P_{tea}$ and injected into subsequent HPGFIM. Given the lightweight feature extractor defined by Equation (1) along with its output $X_{i}$, and the progressive prompt $\left(F_{dense},F_{tea}\right)$, HPGFIM aligns them to a shared embedding space and performs prompt-guided interaction fusion based on 2d selective space (SS2D) [37]. Different from common strategies in which existing works treat $X_{i}$ as the main stream and treat other prompts as guidance, without concatenation or attention, we use $X_{i}$ as an explicit conditioning injection throughout the interaction block, repeatedly modulating both $F_{dense}$ and $F_{tea}$ prompts before and after SS2D, as well as during residual refinement.

Let $C_{i}$ denote the transformed conditioning feature from $X_{i}$. We first inject $C_{i}$ into both branches before the SS2D interaction:

(11) $$\begin{array}{c} U=F_{dense}+C_{i} \end{array}$$

(12) $$\begin{array}{c} V=F_{tea}+C_{i}. \end{array}$$

Then, we apply a bidirectional SS2D operator where the scan parameters of one branch are generated from the other branch, realizing a prompt-guided cross interaction:

(13) $$\begin{array}{c} Y_{dense}=\mathrm{SS}2D\;\left(U;\;\theta (V)\right) \end{array}$$

(14) $$\begin{array}{c} Y_{tea}=\mathrm{SS}2D\;\left(V;\;\theta (U)\right) \end{array}$$

where θ(·) denotes the SS2D parameterization function (e.g., producing Δ,A,B,C,D used by a selective scan).

After SS2D, $C_{i}$ is injected again to reinforce stage-aware conditioning:

(15) $$\begin{array}{c} Y_{dense}\leftarrow Y_{dense}+C_{i} \end{array}$$

(16) $$\begin{array}{c} Y_{tea}\leftarrow Y_{tea}+C_{i} \end{array}$$

We compute branch-specific gates to modulate the interacted responses:

(17) $$\begin{array}{c} g_{dense}=\sigma \;\left(\mathrm{SiLU}\;\left(\mathrm{Linear}\left(F_{dense}\right)\right)\right) \end{array}$$

(18) $$\begin{array}{c} g_{tea}=\sigma \;\left(\mathrm{SiLU}\;\left(\mathrm{Linear}\left(F_{tea}\right)\right)\right) \end{array}$$

and we apply gating to obtain the outputs of the interaction core:

(19) $$\begin{array}{c} O_{dense}=\mathrm{Linear}\;\left(Y_{dense}⊙g_{dense}\right) \end{array}$$

(20) $$\begin{array}{c} O_{tea}=\mathrm{Linear}\;\left(Y_{tea}⊙g_{tea}\right) \end{array}$$

Finally, we apply residual updates and an MLP refinement, where $C_{i}$ is injected multiple times to stabilize the fusion:

(21) $$\begin{array}{c} H_{dense}=O_{dense}+F_{dense} \end{array}$$

(22) $$\begin{array}{c} H_{tea}=O_{tea}+F_{tea} \end{array}$$

(23) $$\begin{array}{c} F_{dense}^{\prime }=H_{dense}+C_{i}+\mathrm{MLP}\;\left(\mathrm{LN}(H_{dense}+C_{i})\right)+C_{i} \end{array}$$

(24) $$\begin{array}{c} F_{tea}^{\prime }=H_{tea}+C_{i}+\mathrm{MLP}\;\left(\mathrm{LN}(H_{tea}+C_{i})\right)+C_{i} \end{array}$$

The refined features $F_{dense}^{\prime }$ and $F_{tea}^{\prime }$ are then concatenated and projected to form the fused representation $F^{\prime }$ for decoding.

### 3.4. Dynamic Selective-Frequency Decoder (DSFD)

Accurate segmentation requires both low-frequency global consistency (shape/region) and high-frequency boundary precision. Static frequency operations may fail under varying noise levels and target morphologies. We thus design DSFD to dynamically select frequency transformations via a frequency-decomposed gating mechanism and explicitly exploit the complementarity between low and high frequencies.

**Figure 2:**
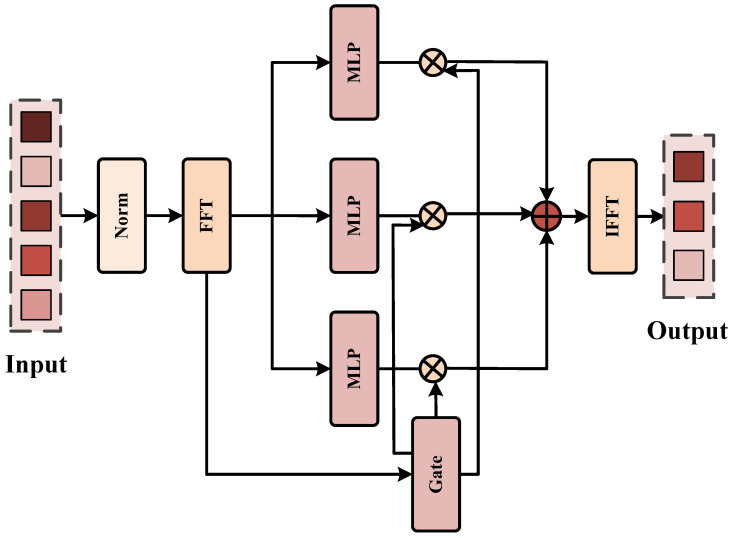
Architecture of the dynamic selective-frequency decoder (DSFD). The input feature map is first normalized and transformed to the frequency domain via FFT. Three parallel MLP branches extract multi-scale frequency features, which are then fused with learnable gates to suppress redundant noise. Finally, the fused frequency features are converted back to the spatial domain via IFFT to generate the refined segmentation output.

Let $X_{i}\in R^{C\times H_{i}\times W_{i}}$ be the input to ${\mathrm{DSD}}_{i}$. We first normalize and reshape it into spatial tokens $T_{i}\in R^{N_{i}\times C}$ with $N_{i}=H_{i}W_{i}$, and we then transform the tokens into the frequency domain as shown in Figure 2:

(25) $$\begin{array}{c} {\bar{T}}_{i}=\mathrm{Norm}\left(T_{i}\right) \end{array}$$

(26) $$\begin{array}{c} F_{i}=F\left({\bar{T}}_{i}\right) \end{array}$$

where F(·) denotes the FFT operation applied along the token axis. As illustrated, the frequency tokens $F_{i}$ are fed into *K* parallel MLP branches:

(27) $$Z_{i}^{\left(k\right)}=\phi_{k}\left(F_{i}\right),\;k=1,\ldots ,K$$

where each $\phi_{k}\left(\cdot \right)$ represents an MLP operating in the frequency domain, providing diverse learnable frequency transformations. A lightweight gating network takes the same frequency representation $F_{i}$ and generates selection weights over all branches:

(28) $$\alpha_{i}=\mathrm{softmax}\left(g(F_{i})\right)$$

Each branch output is modulated by its corresponding gate weight (the ⊗ blocks in the figure) and then aggregated via the ⊕ node:

(29) $${\tilde{F}}_{i}=\sum_{k=1}^{K}\alpha_{i}^{\left(k\right)}⊙Z_{i}^{\left(k\right)}$$

The aggregated frequency tokens are converted back to the token domain via IFFT and reshaped into final feature maps:

(30) $$\begin{array}{c} {\tilde{T}}_{i}=F^{-1}\left({\tilde{F}}_{i}\right) \end{array}$$

(31) $$\begin{array}{c} R_{i}={\mathrm{reshape}}^{-1}\left({\tilde{T}}_{i}\right) \end{array}$$

After obtaining $R_{1},R_{2},R_{3},R_{4}$, we align $R_{2},R_{3},R_{4}$ to the spatial size of $R_{1}$ by interpolation and concatenate them along the channel dimension to form the middle representation:

(32) $$M_{\mathrm{res}}=\mathrm{Concat}\left(\mathrm{Up}\left(R_{2}\right),\;\mathrm{Up}\left(R_{3}\right),\;\mathrm{Up}\left(R_{4}\right)\right),$$

(33) $$F=\mathrm{Fuse}\left(R_{1},\;M_{\mathrm{res}}\right).$$

We then combine middleres with the finest-scale decoded feature $R_{1}$ to obtain the final decoded feature *F*. Finally, DSFD predicts a mask and upsamples it to the original resolution:

(34) $$\hat{Y}=\mathrm{Up}\left(\mathrm{SegHead}(F)\right)$$

### 3.5. Training Objective

The overall training objective consists of a standard segmentation loss, including IoU loss and BCE loss.

(35) $$L=L_{IoU}+\lambda \;L_{BCE},$$

where λ balances loss consistency. The IoU increases the contribution of pixels that are difficult to predict by assigning them larger weights, encouraging the model to concentrate on structurally important regions in challenging cases. In addition, the BCE provides complementary pixel-wise supervision with the same weighting principle. Together, these two terms promote both region-level overlap quality and fine-grained discrimination.

## 4. Experiment

### 4.1. Experiment Settings

The setting of pretrained models of the LFE and PPTB module we use are SAM2 Hiera [27] and [38], respectively. All the experiments are conducted in Ubuntu 20.04, NVIDIA 4090 GPU, Pytorch 2.1. Overall, six benchmark datasets are used for evaluations, including common carotid artery ultrasound images (CCAUI) [39], 2D Cardiac ultrasound dataset (CAMUS) [40], thyroid ultrasound dataset (DDTI) [41], fetal head ultrasound dataset (HC-18) [42], fetal head–pubic symphysis (JNU-LFM) [43], and thyroid nodule image dataset (TN3K) [44]. To comprehensively evaluate the effectiveness of our method, all experiments employ 5-fold cross-validation. To ensure experimental integrity, we re-run all comparative experiments in the same experimental environment and report them as mean ± standard deviation (SD). Code is available at https://github.com/Ezrealone/RPFeaNet (accessed on 2 March 2026). In detail, all images among the dataset we use are resized into 256 × 256. For transformation, we only use vertical and horizontal flips. The number of epochs is 70, and batch size is 12. The AdamW optimizer is selected with 1 × $10^{-4}$. λ used in the loss function is 0.1, and a IoU loss is weighted by a focal parameter of 2. These basic settings are uniformly applied to all comparative methods. To verify the statistical significance of the performance improvement of our method over comparative baselines, paired *t*-tests are conducted based on the results of 5-fold cross-validation. The null hypothesis is that there is no significant difference between the performance of our proposed RPFeaNet method and the comparison method. A *p*-value of less than 0.05 (*p* < 0.05) is considered statistically significant, which is marked with an asterisk (∗) in the table to indicate that the baseline method is significantly inferior to our proposed method.

### 4.2. Comparative Methods

Extensive experiments are conducted on these six benchmark datasets to validate the effectiveness of the proposed RPFeaNet method. To comprehensively evaluate its segmentation performance, we compare our approach with nine state-of-the-art (SOTA) ultrasound image segmentation methods.

- U-Net [45] enabled accurate pixel-wise segmentation by combining contextual information and precise localization via multi-scale skip connections.
- UNet++ [46] designed a method for medical image semantic and instance segmentation based on redesigned skip connections and multi-depth network ensemble.
- AttUNet [47] boosted medical image segmentation accuracy by suppressing irrelevant background features and highlighting salient target organ features.
- nnU-Net [48] integrated fixed parameters, interdependent rules and empirical decisions to realize automated preprocessing, network construction, training and post-processing.
- Swin-Unet [49] integrated Swin Transformer blocks and symmetric encoder-decoder structure to model global contextual and multi-scale hierarchical features.
- TransFuse [50] captured global dependencies and low-level spatial details via parallel CNN and Transformer branches.
- H2Former [51] integrated CNNs, multi-scale channel attention and Transformers to capture long-range dependencies, fuse multi-scale features and ensure computational efficiency for medical image segmentation.
- ScribFormer [52] enabled high-performance scribble-based medical image segmentation by capturing global shape information and fusing local and global features via a triple-branch structure comprising a CNN, Transformer and attention-guided class activation map.
- LGFFM [35] for ultrasound image segmentation integrated a Parallel Bi-Encoder, frequency-domain mapping module and multi-domain fusion to capture local–global and frequency-domain features, addressing low resolution, noise and poor generalization of existing methods.

To comprehensively evaluate the segmentation performance of the proposed method on the seven evaluation metrics, including Jaccard similarity coefficient (Jaccard), Dice similarity coefficient (Dice), weighted F-measure [53], S-measure [54], mean E-measure [55], average symmetric surface distance (ASSD), and mean square error (MSE), they were adopted to quantify region overlap, structural similarity, boundary proximity, and pixel-wise consistency from multiple perspectives. Notably, $F_{\beta }^{w}$ represents the weighted F-measure, $S_{\alpha }$ represents the S-measure, and $E_{\phi }$ represents the mean E-measure.

### 4.3. Result Analysis

#### 4.3.1. The Results of CCAUI and CAMUS

**Figure 3:**
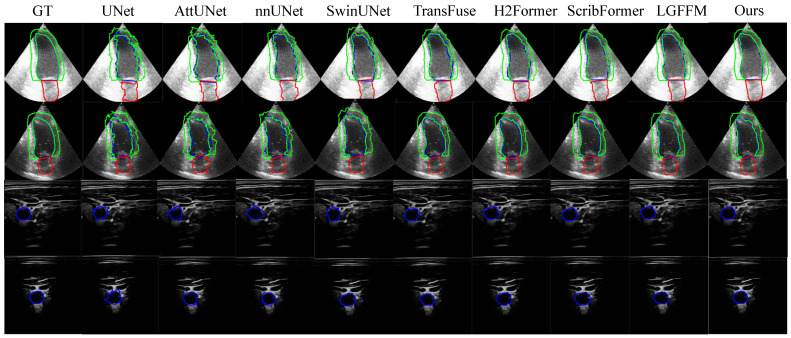
Visual segmentation results on CCAUI and CAMUS datasets. Top two rows belong to the CAMUS dataset, and the bottom two rows belong to the CCAUI dataset. The proposed method demonstrates improved segmentation accuracy and boundary consistency compared to state-of-the-art methods, including UNet, AttUNet, nnUNet, SwinUNet, TransFuse, H2Former, ScribFormer, and LGFFM.

To evaluate the superiority of our proposed method RPFeaNet with states-of-the-art methods, the results of the CCAUI and CAMUS datasets can be found in Table 2. The visualized result can be found in Figure 3, which clearly demonstrates that our proposed method achieves more accurate and visually smooth segmentation contours on the CAMUS dataset with three classes. Both datasets focus on cardiac ultrasound imaging, where the segmented targets exhibit similar statistical shape and size characteristics. Quantitative results clearly demonstrate that our proposed RPFeaNet achieves an average performance gain of 10% across the Jaccard, Dice, ASSD and MSE metrics.

In the CCAUI dataset, the model delivers a substantial improvement over the state-of-the-art LGFFM and other Transformer-based methods with the Jaccard and Dice coefficients rising by 16.7% and 8.3%, respectively, the ASSD metric dropping by 58.9%, and the MSE metric decreasing by 62.2%. This significant enhancement fully validates the model’s superior ability in capturing target regions and fitting boundary details for ultrasound images with severe noise and blurred edges. Notably, RPFeaNet maintains the smallest standard deviation (SD) across all metrics (Jaccard SD = 0.0062), demonstrating superior stability in handling noisy ultrasound data. This significant enhancement fully validates the model’s ability to accurately capture target regions and fit boundary details under adverse imaging conditions. For the CAMUS dataset, even the basic U-Net model can achieve a Dice coefficient of 91%. Our RPFeaNet achieves steady and effective optimization. It outperforms the second one with slight increases in the Jaccard and Dice coefficients, and achieves the optimal value of the mean E-measure metric among all comparison methods. RPFeaNet maintains competitive performance with significantly higher core segmentation metrics. The model’s ability to break through the performance benefits from its targeted architectural design for the inherent characteristics of ultrasound images. The progressive prompt generation module provides hierarchical spatial prior guidance, making up for the deficiency of traditional models that rely solely on pixel-level supervision. The high-level prompt-guided feature interaction module strengthens the capture of target shape and texture features through cross-interaction fusion. The dynamic selective-frequency decoder achieves precise separation and fusion of high- and low-frequency information, effectively improving the accuracy of boundary segmentation.

#### 4.3.2. The Results of DDTI and TN3K

**Figure 4:**
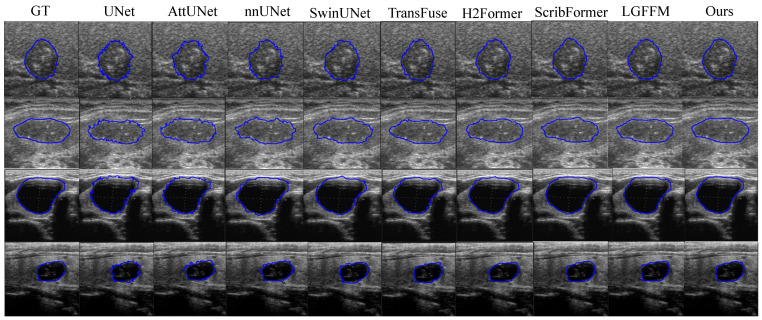
Visual segmentation results on DDTI and TN3K datasets. The top two rows belong to the DDTI dataset, and the bottom two rows belong to the TN3K dataset. The proposed method demonstrates improved segmentation accuracy and boundary consistency compared to state-of-the-art methods, including UNet, AttUNet, nnUNet, SwinUNet, TransFuse, H2Former, ScribFormer, and LGFFM.

The DDTI and TN3K datasets are analyzed together as they are both thyroid ultrasound image benchmarks. Specifically, DDTI focuses on larger targets, while TN3K targets small nodules. TN3K presents greater challenges due to its more blurred nodule boundaries, making it a critical benchmark for verifying the robustness of segmentation models. Quantitative results demonstrate that our proposed RPFeaNet maintains a consistent and significant performance advantage over state-of-the-art comparison methods on both thyroid ultrasound datasets, as shown in Table 3 and Figure 4.

On the DDTI dataset, where most comparison methods have already achieved relatively satisfactory segmentation performance, RPFeaNet still achieves steady and refined optimization compared with the state-of-the-art (SOTA) method. The Jaccard and Dice coefficients are slightly improved, the ASSD metric is reduced by 11.0% (from 0.4760 to 0.4235), and the MSE metric drops by 2.8% (from 0.0461 to 0.0448). The segmentation results on the TN3K dataset more prominently reflect the core advantages of RPFeaNet: it achieves an all-round performance breakthrough compared with other methods. The Jaccard and Dice coefficients are increased by 1.4% and 1.2%, respectively, maintaining the stability of regional segmentation in complex scenarios. The ASSD metric is drastically reduced by 21.1% (from 2.4380 to 1.9237). This fully demonstrates that the dynamic selective-frequency decoder of RPFeaNet can accurately capture the high-frequency information of blurred small nodule boundaries and effectively suppress the interference of ultrasound noise on boundary segmentation.

#### 4.3.3. The Results of HC-18 and JNU-IFM

**Figure 5:**
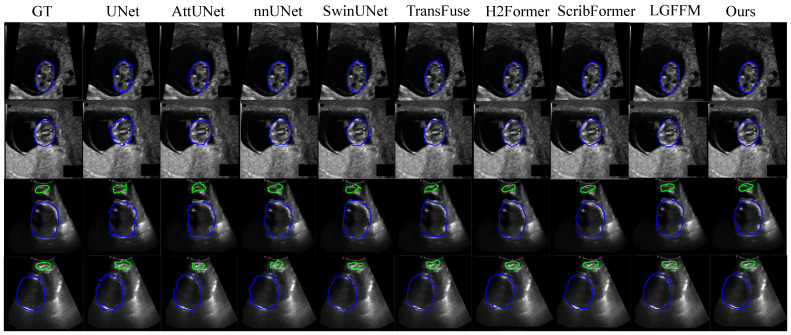
Visual segmentation results on HC-18 and JNU-IFM datasets. The top two rows belong to the HC-18 dataset, and the bottom two rows belong to the JNU-IFM dataset. The proposed method demonstrates improved segmentation accuracy and boundary consistency compared to state-of-the-art methods, including UNet, AttUNet, nnUNet, SwinUNet, TransFuse, H2Former, ScribFormer, and LGFFM.

The HC-18 and JNU-IFM datasets are both designed for fetal ultrasound image segmentation, suffering from severe ultrasound artifacts, low contrast between adjacent tissues, and irregular target boundaries, leading to significantly increased segmentation difficulty. Quantitative results in Table 4 and visualization results in Figure 5 show that our proposed RPFeaNet achieves consistent and outstanding performance advantages over all comparison methods on both fetal ultrasound datasets.

On the HC-18 dataset, most comparison methods already achieve high overall segmentation performance due to the regular fetal head shape. For Jaccard and Dice coefficients, RPFeaNet outperforms others by 0.09 to 0.1. The $S_{\alpha }$ and $E_{\phi }$ metrics are also slightly improved, and the MSE metric is optimized to 0.0458, maintaining competitive segmentation accuracy. Notably, the ASSD metric of RPFeaNet is only slightly higher than that of other methods. The segmentation results on the JNU-IFM dataset demonstrate the core advantages of RPFeaNet in handling complex fetal ultrasound structures. While all comparison methods experience significant performance degradation on these datasets, RPFeaNet achieves comprehensive improvements. The Jaccard and Dice coefficients are increased by 5.4% and 3.6%, respectively, representing a substantial leap in regional segmentation accuracy for complex anatomical structures. The ASSD metric also shows remarkable improvement. This fully demonstrates that the progressive prompt generation module of RPFeaNet can effectively capture spatial prior information of complex fetal head–pubic symphysis structures via progressive guidance. Furthermore, the $S_{\alpha }$ metric of RPFeaNet reaches 0.8947, and the MSE metric is optimized to 0.0527, achieving the best performance among all comparison methods. These results fully validate the model’s superior capability in preserving structural similarity and reducing pixel-level errors for complex fetal ultrasound segmentation.

#### 4.3.4. Complexity Analysis

To provide a more comprehensive assessment of our method, we perform a complexity analysis, as summarized in Table 5. The table reports key metrics, including inference speed (frames per second, FPS), number of parameters (M), and floating-point operations (FLOPs). Compared with lightweight Transformer-based models, our FPS is relatively lower, which is reasonable since our framework used SS2D blocks in HPGFIM rely on Mamba’s selective scan, which involves sequential feature processing (less parallelizable than CNN convolutions/Transformer attention). Our RPFeaNet maintains competitive FPS, indicating strong practical applicability. This advantage primarily comes from the adopted lightweight architectural design. In addition, our model achieves balanced computational efficiency, making it suitable for real-world deployment.

### 4.4. Ablation Studies

To thoroughly validate the contribution of each component in RPFeaNet, we conducted ablation studies on the CCAUI and CAMUS datasets. We establish a baseline model that utilizes the same backbone and segmentation head but replaces our proposed modules with a simple U-Net decoder. Based on this, we progressively incorporated the progressive prompt generation module (PPGM), high-level prompt-guided feature interaction module (HPGFIM), and dynamic selective-frequency decoder (DSFD) to analyze their individual and combined impacts compared with our RPFeaNet.

The quantitative results are reported in Table 6 and Table 7. Comparing the first row, the inclusion of the PPGM yields a substantial improvement. This indicates that the progressive prompt guidance strategy effectively suppresses ultrasound-specific noise and compacts the representation into cleaner feature manifolds, providing a more robust initial feature basis for the decoder. The second row further boosts performance. This significant gain validates that the prompt-guided feature interaction mechanism successfully mitigates the deficiency of insufficient feature discriminability in ultrasound images by dynamically recalibrating the weights of multi-level prompts and backbone features, ensuring that complementary spatial information is fully exploited. The inclusion of the DSFD refines the segmentation results in last row, reaching an optimal Dice value of 93.0% on CCAUI and 95.7% on CAMUS. This demonstrates that the our final model precisely delineating the target boundaries in ultrasound images.

Furthermore, we provide the ablation of sub-branches within core module. The ablation results in Table 8 clearly demonstrate the effectiveness of each sub-branch (LEF, PPTB, PPSB) and their effects on the CCAUI dataset. Specifically, among the single sub-branch configurations, the PPTB sub-branch achieves the best performance with a Dice coefficient of 0.9083 and an ASSD of 0.2089, outperforming LEF (Dice: 0.9015, ASSD: 0.2217) and PPSB (Dice: 0.8957, ASSD: 0.2356) individually. When combining two sub-branches, the PPTB + PPSB combination yields better results than LEF + PPTB and LEF + PPSB, indicating a stronger complementary effect between PPTB and PPSB. Notably, the full configuration integrating all three sub-branches (LEF + PPTB + PPSB) achieves the optimal performance across all metrics, with the highest Dice (0.9300) and Jaccard (0.8783) coefficients, as well as the lowest ASSD (0.1507). These results confirm that each sub-branch contributes positively to the model’s performance.

### 4.5. Limitations

Here, we discuss the inherent limitations of our work. In terms of generalization, while the model is trained and validated on mainstream ultrasound modalities (including carotid, cardiac, fetal, and thyroid imaging), its performance remains insufficiently verified for niche clinical use cases and underrepresented imaging scenarios. Additionally, the fixed 256 × 256 input size requires resizing of clinical high-resolution ultrasound images, potentially losing fine-grained details of small lesions critical for clinical diagnosis. The Mamba-based SS2D blocks in HPGFIM use sequential selective scan operations, which are parameter-efficient but less parallelizable than CNN convolutions or Transformer self-attention. These limitations highlight critical directions for future work to enhance the model’s practical value for clinical deployment and real-world applicability.

## 5. Conclusions

This paper presents a medical ultrasound image segmentation framework (RPFeaNet). This model effectively mitigates the impact of ultrasound-specific noise and low contrast and significantly improves segmentation accuracy and boundary fitting by progressive prompt generation, high-level feature interaction fusion, and a dynamic frequency decoder. Experimental results on multiple datasets show that RPFeaNet outperforms existing SOTA methods. In future work, we will further apply the model for real clinical cases and extend it to multi-modal ultrasound image segmentation tasks.

## Figures and Tables

**Table 1:** Comparison of RPFeaNet with representative state-of-the-art ultrasound segmentation methods (focused on prompt application, prior modeling and frequency processing).

Method	Venue	Prompt Application Position	Prior Modeling Strategy	Frequency-Domain Processing
APG-SAM [24]	ESWA 2025	Box Prompt, Point Prompt	Yolov9 Detection	-
CC-SAM [22]	ECCV 2024	LLM Text Prompt	Grounding DINO	No frequency-domain processing
FreqDINO [23]	ArXiv 2025	Spatial Semantic Prompt	DINOv3	Fixed high-/low-frequency fusion
LGFFM [35]	IEEE TMI 2025	-	SAM2	Static Wavelet frequency fusion
RPFeaNet (Ours)	-	Low-to-high level progressive prompt	Structural (CLIP), patch-level encoder, semantic (SAM2)	Prompt-guided dynamic selective-frequency fusion

**Table 2:** Quantitative comparison of different segmentation methods on CCAUI and CAMUS datasets (mean ± SD, *: *p* < 0.05).

Dataset	Method	Jaccard	Dice	$F_{\beta }^{w}$	$S_{\alpha }$	$E_{\phi }$	ASSD	MSE
CCAUI	U-Net [45]	0.7025 ± 0.0123 *	0.8214 ± 0.0095 *	0.9856 ± 0.0032 *	0.9512 ± 0.0076 *	0.8023 ± 0.0142 *	0.4258 ± 0.0215 *	0.0095 ± 0.0012 *
	UNet++ [46]	0.7218 ± 0.0108 *	0.8386 ± 0.0087 *	0.9879 ± 0.0028 *	0.9587 ± 0.0069 *	0.8195 ± 0.0131 *	0.3964 ± 0.0198 *	0.0088 ± 0.0010 *
	AttUNet [47]	0.7352 ± 0.0095 *	0.8491 ± 0.0076 *	0.9892 ± 0.0025 *	0.9623 ± 0.0062 *	0.8317 ± 0.0118 *	0.3725 ± 0.0182 *	0.0081 ± 0.0009 *
	nnU-Net [48]	0.7486 ± 0.0089 *	0.8593 ± 0.0071 *	0.9905 ± 0.0022 *	0.9668 ± 0.0058 *	0.8429 ± 0.0109 *	0.3682 ± 0.0175 *	0.0083 ± 0.0009 *
	Swin-Unet [49]	0.7154 ± 0.0112 *	0.8327 ± 0.0092 *	0.9871 ± 0.0030 *	0.9564 ± 0.0072 *	0.8136 ± 0.0135 *	0.4059 ± 0.0203 *	0.0091 ± 0.0011 *
	TransFuse [50]	0.7289 ± 0.0102 *	0.8435 ± 0.0082 *	0.9885 ± 0.0026 *	0.9605 ± 0.0065 *	0.8258 ± 0.0125 *	0.3847 ± 0.0190 *	0.0085 ± 0.0010 *
	H2Former [51]	0.7395 ± 0.0091 *	0.8526 ± 0.0074 *	0.9898 ± 0.0024 *	0.9641 ± 0.0060 *	0.8362 ± 0.0115 *	0.3659 ± 0.0172 *	0.0080 ± 0.0008 *
	ScribFormer [52]	0.7451 ± 0.0087 *	0.8572 ± 0.0070 *	0.9912 ± 0.0021 *	0.9675 ± 0.0057 *	0.8405 ± 0.0110 *	0.3671 ± 0.0173 *	0.0082 ± 0.0009 *
	LGFFM [35]	0.7525 ± 0.0085 *	0.8586 ± 0.0068 *	0.9923 ± 0.0020 *	0.9736 ± 0.0052 *	0.8586 ± 0.0105 *	0.3666 ± 0.0170 *	0.0082 ± 0.0009 *
	RPFeaNet (Ours)	0.8783 ± 0.0062	0.9300 ± 0.0045	0.9967 ± 0.0015	0.9879 ± 0.0038	0.9300 ± 0.0065	0.1507 ± 0.0085	0.0031 ± 0.0005
CAMUS	U-Net [45]	0.9093 ± 0.0026 *	0.9185 ± 0.0021 *	0.8512 ± 0.0030 *	0.9215 ± 0.0019 *	0.2216 ± 0.0041 *	0.2854 ± 0.0053 *	0.0356 ± 0.0012 *
	UNet++ [46]	0.9101 ± 0.0017 *	0.9324 ± 0.0015 *	0.8726 ± 0.0022 *	0.9308 ± 0.0014 *	0.2305 ± 0.0035 *	0.2469 ± 0.0044 *	0.0321 ± 0.0010 *
	AttUNet [47]	0.9079 ± 0.0032 *	0.9432 ± 0.0028 *	0.8915 ± 0.0035 *	0.9386 ± 0.0026 *	0.2387 ± 0.0039 *	0.2158 ± 0.0038 *	0.0298 ± 0.0009 *
	nnU-Net [48]	0.9207 ± 0.0056 *	0.9506 ± 0.0049 *	0.9058 ± 0.0051 *	0.9459 ± 0.0045 *	0.2423 ± 0.0042 *	0.1987 ± 0.0035 *	0.0292 ± 0.0008 *
	Swin-Unet [49]	0.8751 ± 0.0017 *	0.9397 ± 0.0020 *	0.8864 ± 0.0024 *	0.9412 ± 0.0018 *	0.2365 ± 0.0037 *	0.2241 ± 0.0040 *	0.0305 ± 0.0009 *
	TransFuse [50]	0.8589 ± 0.0109 *	0.9461 ± 0.0063 *	0.8967 ± 0.0071 *	0.9438 ± 0.0058 *	0.2401 ± 0.0045 *	0.2095 ± 0.0039 *	0.0296 ± 0.0009 *
	H2Former [51]	0.9182 ± 0.0076 *	0.9528 ± 0.0052 *	0.9102 ± 0.0058 *	0.9486 ± 0.0048 *	0.2445 ± 0.0043 *	0.1896 ± 0.0033 *	0.0289 ± 0.0008 *
	ScribFormer [52]	0.9055 ± 0.0024 *	0.9552 ± 0.0026 *	0.9185 ± 0.0029 *	0.9507 ± 0.0024 *	0.2469 ± 0.0040 *	0.1789 ± 0.0031 *	0.0287 ± 0.0007 *
	LGFFM [35]	0.9204 ± 0.0041 *	0.9559 ± 0.0023 *	0.9204 ± 0.0025 *	0.9518 ± 0.0021 *	0.2482 ± 0.0038 *	0.1716 ± 0.0029 *	0.0288 ± 0.0006 *
	RPFeaNet (Ours)	0.9216 ± 0.0025	0.9565 ± 0.0018	0.9217 ± 0.0020	0.9519 ± 0.0017	0.2488 ± 0.0032	0.1778± 0.0030	0.0291 ± 0.0007

**Table 3:** Quantitative comparison of different segmentation methods on DDTI and TN3K datasets (mean ± SD, *: *p* < 0.05).

Dataset	Method	Jaccard	Dice	$F_{\beta }^{w}$	$S_{\alpha }$	$E_{\phi }$	ASSD	MSE
DDTI	U-Net [45]	0.7625 ± 0.0123 *	0.8614 ± 0.0095 *	0.9456 ± 0.0032 *	0.8712 ± 0.0076 *	0.8123 ± 0.0142 *	0.5258 ± 0.0215 *	0.0495 ± 0.0012 *
	UNet++ [46]	0.7818 ± 0.0108 *	0.8786 ± 0.0087 *	0.9579 ± 0.0028 *	0.8887 ± 0.0069 *	0.8295 ± 0.0131 *	0.4964 ± 0.0198 *	0.0488 ± 0.0010 *
	AttUNet [47]	0.7952 ± 0.0095 *	0.8891 ± 0.0076 *	0.9692 ± 0.0025 *	0.8923 ± 0.0062 *	0.8417 ± 0.0118 *	0.4725 ± 0.0182 *	0.0481 ± 0.0009 *
	nnU-Net [48]	0.8086 ± 0.0089 *	0.8993 ± 0.0071 *	0.9705 ± 0.0022 *	0.8968 ± 0.0058 *	0.8529 ± 0.0109 *	0.4682 ± 0.0175 *	0.0483 ± 0.0009 *
	Swin-Unet [49]	0.7754 ± 0.0112 *	0.8727 ± 0.0092 *	0.9571 ± 0.0030 *	0.8864 ± 0.0072 *	0.8236 ± 0.0135 *	0.5059 ± 0.0203 *	0.0491 ± 0.0011 *
	TransFuse [50]	0.7889 ± 0.0102 *	0.8835 ± 0.0082 *	0.9685 ± 0.0026 *	0.8905 ± 0.0065 *	0.8358 ± 0.0125 *	0.4847 ± 0.0190 *	0.0485 ± 0.0010 *
	H2Former [51]	0.8095 ± 0.0091 *	0.8926 ± 0.0074 *	0.9798 ± 0.0024 *	0.8941 ± 0.0060 *	0.8462 ± 0.0115 *	0.4659 ± 0.0172 *	0.0480 ± 0.0008 *
	ScribFormer [52]	0.8151 ± 0.0087 *	0.8972 ± 0.0070 *	0.9812 ± 0.0021 *	0.8975 ± 0.0057 *	0.8505 ± 0.0110 *	0.4671 ± 0.0173 *	0.0482 ± 0.0009 *
	LGFFM [35]	0.8502 ± 0.0085 *	0.9174 ± 0.0068 *	0.9539 ± 0.0020 *	0.9007 ± 0.0052 *	0.9174 ± 0.0105 *	0.4760 ± 0.0170 *	0.0461 ± 0.0009 *
	RPFeaNet (Ours)	0.8536 ± 0.0062	0.9198 ± 0.0045	0.9555 ± 0.0015	0.9021 ± 0.0038	0.9198 ± 0.0065	0.4235 ± 0.0085	0.0448 ± 0.0005
TN3K	U-Net [45]	0.6276 ± 0.0171 *	0.7298 ± 0.0164 *	0.9285 ± 0.0032 *	0.8815 ± 0.0076 *	0.7716 ± 0.0142 *	0.3880 ± 0.0026 *	0.0356 ± 0.0012 *
	UNet++ [46]	0.6424 ± 0.0027 *	0.7487 ± 0.0042 *	0.9324 ± 0.0028 *	0.8908 ± 0.0069 *	0.7805 ± 0.0131 *	0.4070 ± 0.0058 *	0.0321 ± 0.0010 *
	AttUNet [47]	0.6359 ± 0.0161 *	0.7393 ± 0.0164 *	0.9432 ± 0.0025 *	0.8986 ± 0.0062 *	0.7887 ± 0.0118 *	0.4100 ± 0.0070 *	0.0298 ± 0.0009 *
	nnU-Net [48]	0.7102 ± 0.0148 *	0.8062 ± 0.0122 *	0.9506 ± 0.0022 *	0.9059 ± 0.0058 *	0.7923 ± 0.0109 *	0.3700 ± 0.0068 *	0.0292 ± 0.0009 *
	Swin-Unet [49]	0.6599 ± 0.0117 *	0.7576 ± 0.0112 *	0.9397 ± 0.0030 *	0.9012 ± 0.0072 *	0.7865 ± 0.0135 *	0.3420 ± 0.0092 *	0.0305 ± 0.0011 *
	TransFuse [50]	0.7118 ± 0.0118	0.8163 ± 0.0089	0.9461 ± 0.0026	0.9038 ± 0.0065	0.7901 ± 0.0125	0.2820 ± 0.0009	0.0296 ± 0.0010
	H2Former [51]	0.7111 ± 0.0122	0.8072 ± 0.0125	0.9528 ± 0.0024	0.9086 ± 0.0060	0.7945 ± 0.0115	0.2840 ± 0.0067	0.0289 ± 0.0008
	ScribFormer [52]	0.6730 ± 0.0147 *	0.7757 ± 0.0129 *	0.9552 ± 0.0021 *	0.9107 ± 0.0057 *	0.7969 ± 0.0110 *	0.3360 ± 0.0056 *	0.0287 ± 0.0009 *
	LGFFM [35]	0.7243 ± 0.0085 *	0.8210 ± 0.0068 *	0.9693 ± 0.0020 *	0.9331 ± 0.0052 *	0.8265 ± 0.0105 *	0.2438 ± 0.0170 *	0.0297 ± 0.0009 *
	RPFeaNet (Ours)	0.7342 ± 0.0075	0.8306 ± 0.0065	0.9703 ± 0.0015	0.9344 ± 0.0038	0.8142 ± 0.0065	0.1924 ± 0.0027	0.0289 ± 0.0005

**Table 4:** Quantitative comparison of different segmentation methods on HC-18 and JNU-IFM datasets (mean ± SD, *: *p* < 0.05).

Dataset	Method	Jaccard	Dice	$F_{\beta }^{w}$	$S_{\alpha }$	$E_{\phi }$	ASSD	MSE
	U-Net [45]	0.7815 ± 0.0128 *	0.8752 ± 0.0106 *	0.9512 ± 0.0085 *	0.8825 ± 0.0092 *	0.8217 ± 0.0114 *	0.6128 ± 0.0412 *	0.0523 ± 0.0035 *
	UNet++ [46]	0.8028 ± 0.0114 *	0.8906 ± 0.0098 *	0.9635 ± 0.0076 *	0.8947 ± 0.0083 *	0.8389 ± 0.0106 *	0.5814 ± 0.0386 *	0.0501 ± 0.0032 *
	AttUNet [47]	0.8165 ± 0.0107 *	0.8987 ± 0.0092 *	0.9718 ± 0.0069 *	0.9012 ± 0.0078 *	0.8495 ± 0.0099 *	0.5578 ± 0.0364 *	0.0489 ± 0.0030 *
	nnU-Net [48]	0.8302 ± 0.0099 *	0.9075 ± 0.0085 *	0.9786 ± 0.0063 *	0.9085 ± 0.0072 *	0.8603 ± 0.0092 *	0.5492 ± 0.0348 *	0.0485 ± 0.0028 *
	Swin-Unet [49]	0.7946 ± 0.0119 *	0.8834 ± 0.0102 *	0.9589 ± 0.0080 *	0.8893 ± 0.0087 *	0.8312 ± 0.0111 *	0.5947 ± 0.0398 *	0.0512 ± 0.0033 *
	TransFuse [50]	0.8098 ± 0.0103 *	0.8942 ± 0.0088 *	0.9674 ± 0.0071 *	0.8978 ± 0.0079 *	0.8426 ± 0.0098 *	0.5735 ± 0.0357 *	0.0496 ± 0.0029 *
	H2Former [51]	0.8215 ± 0.0094 *	0.9028 ± 0.0081 *	0.9759 ± 0.0060 *	0.9051 ± 0.0068 *	0.8538 ± 0.0087 *	0.5467 ± 0.0331 *	0.0482 ± 0.0027 *
	ScribFormer [52]	0.8279 ± 0.0089 *	0.9061 ± 0.0077 *	0.9803 ± 0.0057 *	0.9097 ± 0.0065 *	0.8584 ± 0.0083 *	0.5481 ± 0.0324 *	0.0484 ± 0.0026 *
	LGFFM [35]	0.8427 ± 0.0076 *	0.9158 ± 0.0064 *	0.9618 ± 0.0051 *	0.9125 ± 0.0058 *	0.8716 ± 0.0072 *	0.5398 ± 0.0298 *	0.0472 ± 0.0023 *
	RPFeaNet (Ours)	0.8519 ± 0.0042	0.9214 ± 0.0035	0.9645 ± 0.0028	0.9158 ± 0.0031	0.8789 ± 0.0039	0.4876 ± 0.0186	0.0458 ± 0.0017
JNU-IFM	U-Net [45]	0.7128 ± 0.0156 *	0.8315 ± 0.0123 *	0.9326 ± 0.0092 *	0.8517 ± 0.0105 *	0.7824 ± 0.0131 *	1.8254 ± 0.1286 *	0.0615 ± 0.0042 *
	UNet++ [46]	0.7345 ± 0.0142 *	0.8478 ± 0.0115 *	0.9418 ± 0.0085 *	0.8639 ± 0.0097 *	0.7987 ± 0.0124 *	1.6872 ± 0.1175 *	0.0589 ± 0.0039 *
	AttUNet [47]	0.7519 ± 0.0133 *	0.8592 ± 0.0108 *	0.9503 ± 0.0078 *	0.8725 ± 0.0091 *	0.8094 ± 0.0118 *	1.5786 ± 0.1063 *	0.0567 ± 0.0036 *
	nnU-Net [48]	0.7684 ± 0.0125 *	0.8687 ± 0.0101 *	0.9578 ± 0.0072 *	0.8802 ± 0.0085 *	0.8189 ± 0.0112 *	1.4963 ± 0.0984 *	0.0559 ± 0.0034 *
	Swin-Unet [49]	0.7427 ± 0.0147 *	0.8536 ± 0.0112 *	0.9465 ± 0.0089 *	0.8684 ± 0.0099 *	0.8031 ± 0.0127 *	1.6348 ± 0.1132 *	0.0578 ± 0.0038 *
	TransFuse [50]	0.7586 ± 0.0137 *	0.8631 ± 0.0105 *	0.9537 ± 0.0075 *	0.8758 ± 0.0088 *	0.8146 ± 0.0121 *	1.5279 ± 0.1035 *	0.0562 ± 0.0037 *
	H2Former [51]	0.7725 ± 0.0121 *	0.8714 ± 0.0097 *	0.9605 ± 0.0069 *	0.8827 ± 0.0082 *	0.8225 ± 0.0109 *	1.4785 ± 0.0957 *	0.0554 ± 0.0033 *
	ScribFormer [52]	0.7798 ± 0.0116 *	0.8759 ± 0.0093 *	0.9632 ± 0.0066 *	0.8859 ± 0.0079 *	0.8271 ± 0.0105 *	1.4692 ± 0.0942 *	0.0551 ± 0.0032 *
	LGFFM [35]	0.7865 ± 0.0108 *	0.8807 ± 0.0089 *	0.9714 ± 0.0059 *	0.8913 ± 0.0074 *	0.8358 ± 0.0098 *	1.4287 ± 0.0896 *	0.0543 ± 0.0030 *
	RPFeaNet (Ours)	0.7982 ± 0.0053	0.8889 ± 0.0041	0.9738 ± 0.0032	0.8947 ± 0.0038	0.8426 ± 0.0046	1.2854 ± 0.0572	0.0527 ± 0.0021

**Table 5:** Complexity analysis of different segmentation methods.

Method	FPS	Params (M)	FLOPs (G)
U-Net	89.45	34.53	65.52
UNet++	51.74	36.63	138.66
AttUNet	87.28	34.88	66.63
nnU-Net	89.63	34.56	65.72
Swin-UNet	148.99	27.15	5.92
TransFuse	115.63	26.17	8.65
H2Former	107.03	33.63	32.25
ScribFormer	59.71	50.42	54.46
LGFFM	32.22	15.02	64.42
RPFeaNet (Ours)	10.54	29.31	108.12

**Table 6:** Performance comparison of ablation study on CCAUI dataset.

PPGM	HPGFIM	DSFD	Dice ↑	Jaccard ↑	ASSD ↓
✓			0.852	0.741	0.321
	✓		0.867	0.763	0.305
		✓	0.881	0.785	0.289
✓	✓		0.905	0.827	0.242
✓		✓	0.912	0.839	0.231
	✓	✓	0.918	0.848	0.224
✓	✓	✓	0.930	0.878	0.151

**Table 7:** Performance comparison of ablation study on CAMUS dataset.

PPGM	HPGFIM	DSFD	Dice ↑	Jaccard ↑	ASSD ↓
✓			0.921	0.853	0.285
	✓		0.934	0.872	0.268
		✓	0.942	0.889	0.251
✓	✓		0.948	0.901	0.224
✓		✓	0.951	0.907	0.216
	✓	✓	0.953	0.912	0.209
✓	✓	✓	0.957	0.922	0.178

**Table 8:** Ablation analysis of sub-branches (LEF, PPTB, PPSB) on CCAUI dataset.

Sub-Branch Setup	Dice ↑	Jaccard ↑	ASSD ↓
w/LEF only	0.9015	0.8207	0.2217
w/PPTB only	0.9083	0.8324	0.2089
w/PPSB only	0.8957	0.8116	0.2356
w/LEF + PPTB	0.9246	0.8598	0.1783
w/LEF + PPSB	0.9192	0.8503	0.1895
w/PPTB + PPSB	0.9297	0.8685	0.1672
w/LEF + PPTB + PPSB (Full)	0.9300	0.8783	0.1507

## Data Availability

The CCAUI dataset can be accessed at https://data.mendeley.com/datasets/d4xt63mgjm/1 (accessed on 24 December 2025). CAMUS can be downloaded from https://www.creatis.insa-lyon.fr/Challenge/camus/databases.html (accessed on 24 December 2025). TN3K can be downloaded from https://www.kaggle.com/datasets/tjahan/tn3k-thyroid-nodule-region-segmentation-dataset (accessed on 24 December 2025). DDTI can be downloaded from https://www.kaggle.com/datasets/dasmehdixtr/ddti-thyroid-ultrasound-images/data (accessed on 24 December 2025). HC-18 can be downloaded from https://hc18.grand-challenge.org (accessed on 24 December 2025). JNU-IFM can be downloaded from https://figshare.com/articles/dataset/JNU-IFM/14371652/2 (accessed on 24 December 2025).
